# Rethinking and strengthening the Global Health Diplomacy through triangulated nexus between policy makers, scientists and the community in light of COVID-19 global crisis

**DOI:** 10.1186/s41256-021-00195-2

**Published:** 2021-04-13

**Authors:** Mohammed AlKhaldi, Nigel James, Vijay Kumar Chattu, Sara Ahmed, Hamza Meghari, Kirsty Kaiser, Carel IJsselmuiden, Marcel Tanner

**Affiliations:** 1 McGill University, Faculty of Medicine, Montreal, Canada; 2 University of Basel, Basel, Switzerland; 3 Swiss Tropical and Public Health Institute, Basel, Switzerland; 4 Department of Public Health, Unit of Health Systems and Policies, Basel, Switzerland; 5 Council on Health Research for Development, COHRED, Geneva, Switzerland; 6 Health Policy & Demography/Department of Health Policy and Administration, The Pennsylvania State University, Pennsylvania, USA; 7 Department of Medicine, Faculty of Medicine, University of Toronto, Toronto, Canada; 8 Institute of International Relations, The University of the West Indies, St .Augustine, Trinidad and Tobago; 9 Faculty of Medicine, School of Physical & Occupational Therapy, McGill University Health Center, Clinical Epidemiology, Montreal, Canada; 10 Centre de recherche interdisciplinaire en réadaptation (CRIR), Constance Lethbridge Rehabilitation Center du CIUSSS du Centre-Ouest-de-l’Île-de-Montréal, Montreal, Canada; 11 Initiative pour le développement de nouvelles technologies et pratiques en réadaptation (INSPIRE), Institut de réadaptation Gingras-Lindsay de Montréal, CIUSSS Centre-Est-de-l’ile-de-Montréal, Montreal, Canada; 12 Great Ormond Street Hospital for Children, NHS Foundation Trust, London, UK; 13 Global Health and Development, Institute for Global Health, University College London UCL, London, UK; 14 Women Deliver Organization, New York, USA; 15 COHRED Africa, Cape Town, South Africa; 16 Epidemiology and Medical Parasitology, University of Basel, Basel, Switzerland

**Keywords:** COVID-19, Global health diplomacy, Vaccine diplomacy, And science diplomacy, Policy

## Abstract

The COVID-19 pandemic is considerably the biggest global health challenge of this modern era. Spreading across all regions of the world, this corona virus disease has disrupted even some of the most advanced economies and healthcare systems. With an increasing global death toll and no near end in sight, questions on the efficacy of global response mechanisms, including the role and relevancy of global health institutions, have emerged. Using a reflexive content analytic approach, this study sheds light on some of these questions, underscoring the disconnect between science, policymaking, and society. Global health funding approaches; politicization of the pandemic, including political blame gaming; mistrust of government and other institutions; and a lack of robust accountability measures are some of the pandemic response obstacles. However, COVID-19 has also presented an opportunity for a collaboration that may potentially solidify global solidarity. A pandemic response built on strategic global health diplomacy, vaccine diplomacy, and science diplomacy can spur both political and economic benefits, advancing development, health security, and justice. The virus thrives and flourishes in face of political divisions and lack of cooperation. While the current global crisis has exacerbated the existing social injustices in societies, national unity and global solidarity is essential to winning the fight against the COVID-19 pandemic.

## Introduction

The COVID-19 pandemic has become a global health catastrophe of colossal proportions, with far-reaching health and economic ramifications. This novel virus has affected all walks of life — rich or poor—presenting a shared vulnerability. But, undeniably, the poor have endured disproportion impact, including an elevated death toll [1, 2]. Some analysts have argued that the current COVID − 19 predicament can be explained by a “catastrophic failures of science-policy interface.” [3] With the increasing global death toll and seemingly unabated virus spread in all parts of the world, the global health machinery has come under a spotlight of scrutiny. Experts and laypeople alike are pondering on how society reached this point and if policymakers and leaders could have done better with this predicament. This article examines two questions: (1) the relevancy of global health institutions—whether current global health framework is appropriately designed to combat public health threats and (2) the factors facilitating or hindering country governments’ effective responses to the COVID-19 disaster. These questions are particularly important, not only to help in curbing the current crisis, but they also highlight the need for strengthening global health systems in the view of increased emerging and reemerging pandemic threats [4]. .The novel coronavirus disease has amplified the need for increased coordination across geographic and political boundaries and stronger collaboration across science, policymaking, and the community at large. Leveraging tools such as Global Health Diplomacy (GHD), Vaccine Diplomacy (VD), and Science Diplomacy (SD) provides an opportunity to rethinking global health dynamics in ways that foster development, health security, justice, and health equity.

Using peer reviewed and gray literature, this article applies a reflexive content analysis approach to examine and interpret the pandemic response flows and highlight the threats to global welfare. The subsequent paragraphs present, first: the COVID-19 global crisis, second: synthesis of thematic content analysis findings, and finally: recommendations for strengthening the nexus of scientific evidence, policymaking, and the community in pandemic response.

## The COVID-19 global crisis

In the wake of a heightened pandemic, the COVID-19 response has been characterized by a misalignment of voices, ambiguity in messaging, downplaying narratives, and an avalanche of disinformation. In some cases, this disinformation on COVID-19 and lack of social services infrastructure such as water availability and decent housing threaten the current scope of COVID-19 mitigation measures. The virus has exploited and capitalized on current global health architecture’s inefficiencies in bridging “bench evidence to policy”. An erroneous view of politics and public health as opponents in some countries has allowed the virus to thrive. In the midst of all this, the community has largely been treated as “passive recipients” rather than active and equal partners, in as far as designing and implementation of mitigation and response measures is concerned. It has not been all gloomy though, the pandemic has also triggered a greater understanding and cooperation within the international scientific community, with researchers from all continents working around the clock exploring potential vaccines, public health measures, therapeutics and enhanced diagnostics among other priorities [5]. A future in which global pandemics are identified early enough, contained and or controlled, calls for reviewing and redesigning the mandate of global health institutions, including the associated financing frameworks, to smoothen translation of scientific evidence to policy, and foster meaningful society involvement for sustainability. As noted by AlKhaldi et al. (2020), the emerging field of health policy and systems research (HPSR) could be useful in application to this modernistic approach [6]. Adopting HPSR spurs not only new thinking towards a transformational renaissance in health systems but also strengthens synergies between policymakers, scientists, and societies to launch radical reform in sectors, disciplines, and existing bodies, especially in fragile and low-resourced countries. In this context, the application and practice of global health diplomacy (GHD) becomes very critical. It enables multiple stakeholders to contribute to the greater health needs of humanity and foster stronger interdisciplinary approaches, promoting negotiations that shape and manage the global policy environment for health [7].

## Global health mandate and associated financing mechanism

World Health Organization (WHO) declared COVID-19 a Public Health Emergency of International Concern (PHEIC) on March 30, 2020 [8]. The expectation was an all “hands on deck”, member states being called upon to ramp up testing, isolation and contact tracing, as the backbone of the global response. Unfortunately, in some cases, the call was met with anti-science sentiments, blame gaming, and stalled by bureaucracy and politicization of the pandemic [9]. The mandate and independence of WHO has been brought to the spotlight, once again. As a technical agency that derives its mandate and funding from member states, WHO risk’s funding losses as economic superpowers engage in blame shifting and name calling. Traditionally these superpowers contribute the largest funding contributions to WHO [10]. Consequently, a dependency on world superpowers for resources to advance outbreak response compromises the effectiveness of global institutions such as WHO. The priorities of major donors are not always going to align with global interests. The poor and vulnerable maybe be left to suffer [11]. Despite the urgency and centrality of a COVID19 vaccine, the European parliament has recently slashed the funding budget towards research, a move that scientists have described as “lack of ambition or political willingness to tackle global health head on” [12]. Also, the US has already pulled out committed funds to the WHO [13]. As the global political climate continues to shift towards nationalism and anti-immigration, a global health financing architecture centered on goodwill of political and economic superpowers is likely to fail.

Financial resource pooling has been key in driving most of the global health initiatives with significant success. Good examples include the Global Fund to fight AIDS, Tuberculosis, and Malaria (Global Fund) and the GAVI alliance on vaccine preventable diseases [14]. Most of the health budgets of the low-resourced countries are poorly resourced and are donor dependent, including funding support for epidemic control and response. This limited domestic investment slows down the efficiency of the “rapid response”. HPSR drives the efforts of reshaping public health systems to strengthen pandemic preparedness and strategies of recovery taking into account evidence-based systems. This could lead to fundamental changes for improving human lives at the three levels of health systems—micro (improving access to treatment), meso (strengthening the resilience and capacity of local health facilities, clinics, and hospitals), and macro (consolidating responses across sectors and throughout an entire state or national system) [15]. Lack of political will and accountability, system inefficiencies and corruption limit poorly resourced countries’ public health financing [15]. Reportedly, in Zimbabwe, the Minister of Heath was recently ousted for embezzling $60 million ear marked for COVID-19 response [16]. In Ghana, officials from one of the main central hospitals are being investigated for siphoning personal protective equipment (PPE) for private resale at a time when more than 2000 frontline line workers have contracted COVID-19 on account of lack of protective gear [17]. In Libya, WHO continues to seek clarity over $351 million designated for COVID-19 response by the Government of National Accord in Tripoli [18].

## Translating global policy guidelines to suit the local context

During the early phase of the pandemic “flattening the curve” became the global response mantra. Many countries instituted national lockdowns and movement restrictions following WHO recommendations [19]. Unfortunately, not all countries have the capacity to implement some of the recommended measures. Some poor and low-resourced countries that implemented “lock-downs” may not be able to recover their economies any time soon and could face exacerbated downstream impacts related to hunger, malnutrition, violence, and mental health [7]. Admittedly, lockdowns suppressed a surge in cases that would otherwise have put a huge strain on health systems in both the global north and global south countries. To avoid countries being driven into a worse off positions economically, after the pandemic, more technical guidance, local context tailored and country level evidence-driven adaptations could have been helpful [20]. Strong economies thrive on the back-bone of viable public health and vice versa [21].

## Role of Global Health diplomacy (GHD) and science diplomacy (SD) in translating global policies

**Fig. 1:**
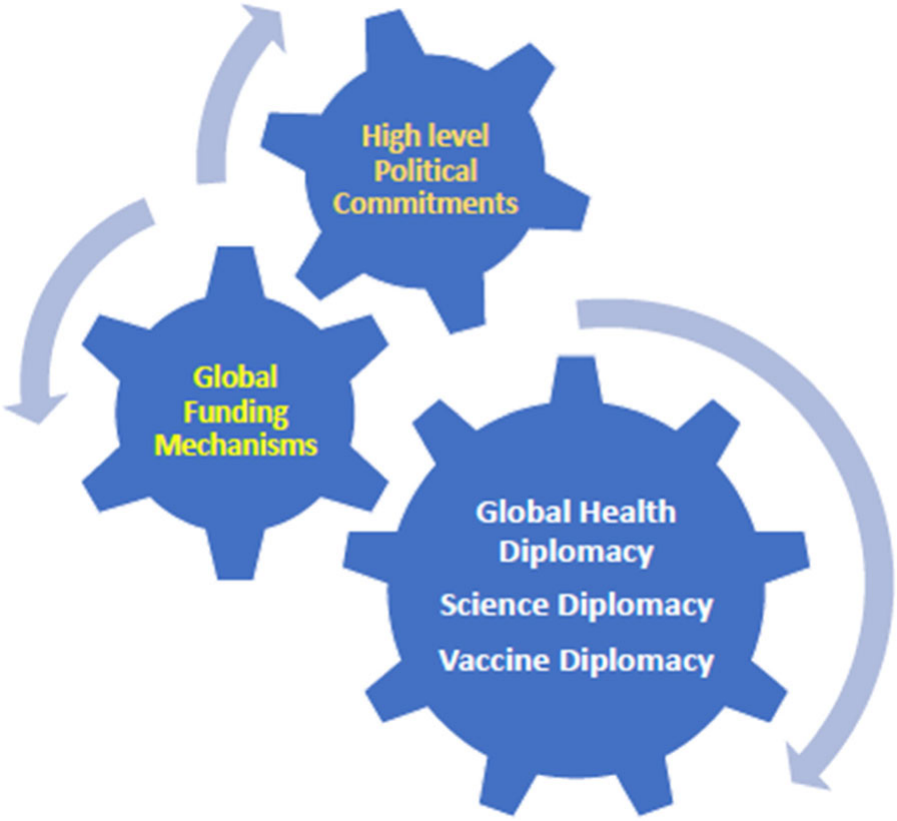
Dynamics between global health diplomacy, political and financial commitments for global development

In order to bring all the global stakeholders on a common platform to fight against this global pandemic, the nations, including both state and non-state country actors, should come together united for a common goal of ensuring a safer world, which is also the goal of GHD. According to WHO, the goal of GHD is primarily to improve health security, population health and the relationship between states. Therefore, investing in and improving health diplomacy will advance collective development, social justice (equity) and national security [5]. Similarly, building bridges between science and policy, and between countries, going beyond geopolitical barriers, is the thrust of Science Diplomacy SD), which is again an essential strategy to overcome COVID-19.It is characterized by international collaboration with science at its core.SD was quite successful and brought an unprecedented global change, as seen by the ozone layer recovery [22]. Unlike the other aspects of medical or public health interventions, vaccines are unique as they are the single most potent intervention developed by humankind. This pandemic is yet another opportunity to prove the efficacy of SD via a potential lifesaving COVID-19 vaccine. Vaccine Diplomacy (VD) is another critical component of GHD, essential to pinning down COVID-19.It brings together organization such as GAVI Alliance, WHO, Gates Foundation, and other global research institutions to ensure global collaboration to accelerate the development and manufacture of COVID-19 vaccines, guaranteeing fair and equitable access of vaccines for every country [23]. A tripartite synergy of GHD, SD and VD, combined with high-level political commitments (heads of states, policy makers), and dedicated funding (to address access, availability and equity issues) has potential to bring to life, effective vaccines, enhancing global health security (Fig. 1). However, to reap the benefits of these ongoing interdisciplinary concepts of diplomatic activities, scientific research community, policymakers and global stakeholders must commit to collective action and commitment for the global development, including supporting the GHD approach in advancing the United Nations Sustainable Development Goals (SDGs).

## Governance and public health messaging

Public health interventions are likely to succeed when the people have trust in their public institutions and systems of government. Whilst some country governments and public institutions are doing well to foster this trust, others still need to pull their sleeves. There has been some reports of data misreporting or underreporting and censorship of information flow [24, 25]. Relatedly, some country goverments have resorted to heavy handed use of military action to enforce lockdowns. In Nairobi, Kenya, 13-year-old boy was hit by a stray bullet fired by police enforcing a stay-at-home order [26]. The situation could be more dire in conflict zones such as Palestine. COVID-19 has revealed major inequalities, fragility, and health insecurity due to prevailing violations of political, socio-economic, and humanitarian rights. This has left the Palestinians living under a multilayered suffering; COVID-19 pandemic, ongoing occupation, and intra-Palestinian divide. Continued raids, arrests, homes demolitions, absence of freedom of movement, lack of control over resources in addition to siege on the Gaza Strip, all impede Palestine’s ability to control the spread of pandemic [6]. General mistrust in government and public institutions, systemic discrimination, and inequalities fueled COVID-19 spread. The “Black Lives Matter” protests that started off in the US and spread to other parts of the world, happened right at the peak of the pandemic, despite “social and physical distancing” recommendations [27, 28]. Protests were a clear message from a disgruntled citizenry that pandemic guidelines come secondary in the face of perceived injustice. The community is likely to follow public health guidelines to the degree they feel, the social contract between themselves and those in power is being upheld. Strategic risk communication tailored community’s to cultural norms and demographic needs is essential in providing information to the public that will equip them with stronger coping mechanisms for COVID-19 and promote adoption of preventive behavoioral practices. The absence of accurate information at community level has been fuelling low risk perception amongst some demographic groups, particularly the young. This low risk perception is dangerous as it promotes risky behaviors, potentially driving the outbreak out of control in some societies. In the US, politicization of COVID-19 messaging between federal and state government slowed down response efforts at the cost of human lives [29]. The pandemic response varied across states and in New York, federal and state governments fought over “who had the responsibility to stock hospitals with ventilators and other personal protective equipment” [30]. The labelling of the pandemic as “Chinese virus” is not only fueling stigma and discrimination against the Chinese American communities [31], but also poisoning global health solidarity [32], in particular when the whole world need China’s assistance to fight against COVID-19. Again, in the US, blacks reported 34% cumulative COVID-19 mortality, despite being only 13.4% of the total population [33]. These examples highlight how intricately linked everyday politics is to pandemic response efforts. Investing in strong and accountable governance systems is an indispensable ingredient for COVID-19 and possible future outbreaks.

## Rethinking and strengthening the nexus of scientific evidence, policy-making and community

As the big powers are fighting for political gains, primacy in marketing vaccines, and furthering their own geo-political agendas on the back of the COVID-19 epidemic, perhaps, it is high time that citizens of the world take back the initiative to ensure health for all. COVID-19 has highlighted the need for enhancing global coordination and collaboration. Going forward, there is a need to a shift to more sustainable financing mechanisms that include stronger private sector participation, especially in developing countries. Other proposals such as the Right to Health Capacity Fund (R2HCF) which aim to break superpower monopoly and promote rights based funding should be fully explored. To achieve this, a civil society-led multi-stakeholder process is essential to create a world, where in or out of health emergency, no one is left behind. Another example is the establishment of the WHO Foundation as an independent grant-making entity that will support the Organization’s efforts to address the most pressing global health challenges. Relatedly, the 2019 Declaration on Universal Health Coverage is a good starting point to a right’s base global pandemic financing mechanism. Additionally, leaders and funders can encourage the use of tools and platforms, such as COHRED’s Research Fairness Initiative (https://rfi.cohred.org) to ensure that collaborations are undertaken in an equitable and impactful way. It is absolutely key that as scientists package their research in a policy relevant and evidence-based fashion, and avoid policy prescriptions or propaganda. Meaningful engagement between public health scientists and the community, even before a pandemic strike builds an environment of trust that is essential for the success of public health interventions. Breaking the silos between science, policy and the community will prevent devastating impacts of pandemics and advance the universal health coverage agenda.

## Conclusion

COVID-19 has brought both challenges and opportunities, and it is crucial to move forward collectively in this multipolar international society with a common goal of ensuring a safer world. HPSR encourages strategic dialogue among three societies (science, politics, and society) and it could strengthen the triangulated nexus by ensuring the needs, priorities, and aspirations of these societies are adequately translated and fulfilled in the age of global health crises. The concepts of global health diplomacy, science diplomacy, vaccine diplomacy and fair research collaboration are the emerging powerful tools, which must be used for unifying the world and in building a safer and interconnected community. UHC and SDGs can only be achieved if the underlying root causes such as socioeconomic, gender and health inequities are appropriately addressed but the current global health crisis has actually exacerbated the existing social injustices in societies. National unity and global solidarity can pose the essence of the triangulated GHD towards achieving considerable progress in global health.

## Data Availability

Not applicable.

## Abbreviations

COVID-19: Novel Coronavirus 2019
WHO: World Health Organization
HPSR: Health Policy and Systems Research
GHD: Global Health Diplomacy
PHEIC: Public Health Emergency of International Concern
AIDS: Acquired Immunodeficiency Syndrome
GAVI: Global Alliance for Vaccines and Immunizations
PPE: Personal Protective Equipment
UNICEF: United Nations Children’s Fund
SD: Science Diplomacy
VD: Vaccine Diplomacy
WEF: World Economic Forum
SDGs: United Nations Sustainable Development Goals
US: United States
R2HCF: Right to Health Capacity Fund
COHRED: Council on Health Research for Development
